# Network Security Situation Prediction Model Based on EMD and ELPSO Optimized BiGRU Neural Network

**DOI:** 10.1155/2022/6031129

**Published:** 2022-06-21

**Authors:** Biao Zhang, Mingqi Jia, Jiazhong Xu, Wanzhao Zhao, Liwei Deng

**Affiliations:** ^1^Heilongjiang Provincial Key Laboratory of Complex Intelligent System and Integration, School of Automation, Harbin University of Science and Technology, Harbin 150080, China; ^2^Guangxi Agricultural Vocational and Technical University, Nanning 530005, Guangxi, China

## Abstract

In order to improve the accuracy of network security situation prediction and the convergence speed of prediction algorithm, this paper proposes a combined prediction model (EMD-ELPSO-BiGRU) based on empirical mode decomposition (EMD) and improved particle swarm optimization (ELPSO) to optimize BiGRU neural network. Firstly, the network security situation data sequence is decomposed into a series of intrinsic mode function by EMD. Then, a particle swarm optimization algorithm (ELPSO) based on cooperative update of evolutionary state judgment and learning strategy is proposed to optimize the hyper-parameters of BiGRU neural network. Finally, a network security situation prediction model based on EMD-ELPSO-BiGRU is constructed to predict each intrinsic mode function, respectively, and the prediction results are superimposed to obtain the final network security situation prediction value. Simulation results show that ELPSO has better optimization performance, and EMD-ELPSO-BiGRU model has higher prediction accuracy and significantly improved convergence speed compared with other traditional prediction methods.

## 1. Introduction

With the rapid development of Internet technology, computer network has become an indispensable means of communication. However, there are various threats in the network environment. Although firewall, intrusion detection system, virus killing, and other technologies have been developed at present, these methods can only deal with the threats and cannot control the overall trend of the network well. Under this background, aiming at the problem of network security, researchers put forward network security situational awareness. Network security situation is a trend of network security situation. According to the change of network environment, network administrators can take measures to avoid network attacks or reduce the damage caused by network attacks. Network security situation prediction is an active defense mechanism [1], which first analyzes and understands the elements of current and past network situation, and then speculates the future network situation. Because the current situation of network security is reflected by the situation value obtained after situation assessment, and the situation value represents the network state value at every moment, the situation prediction problem is actually a time series prediction problem [2]. Because the trend of network security situation change is nonlinear and time-varying, many classical time series prediction methods are difficult to accurately find out the relationship between the current situation of network and the development trend, which leads to the inability to improve the prediction accuracy [3–5].

In the existing research on network security situation prediction, the methods used are mainly divided into three types: mathematical model-based, knowledge-based reasoning, and pattern recognition-based [6].

The method based on mathematical model is the first method applied in network security situation prediction. This method can comprehensively analyze various factors that may affect the change of network security state, construct an evaluation function, and realize the mapping from the set composed of various situation factors to the network security situation space through mathematical expressions. Methods based on mathematical models include analytic hierarchy process, weight analysis, and time series analysis [7]. Wang and Hu [8, 9] predict the network security situation through time series analysis algorithm and analyze multiple historical situation values obtained by situation assessment algorithm in time series to realize the prediction of future network security state. However, because the sliding regression model based on time series requires the input series to meet the stationarity assumption, it cannot always guarantee high accuracy.

In network security situation assessment and prediction, knowledge-based reasoning method uses evidence theory, probability theory, and fuzzy theory to deal with uncertain information that may affect network security, and establishes corresponding assessment and prediction models based on expert knowledge and experience. Yang et al. [10] through the Bayesian algorithm based on probability theory have improved to form a dynamic Bayesian network model, then the prior probability is initialized and the posterior probability is adjusted by combining historical situation data with real-time situation data, and it is successfully applied to network security situation prediction. Yifan [11] proposed a risk assessment method based on Bayesian network, but it cannot be applied to large-scale network environment because of the high cost of calculating joint probability. Ruan [12] applies fuzzy reasoning to situation prediction, describes network security situation based on fuzzy sets, and combines Markov process. At the same time, genetic algorithm is introduced into fuzzy membership function, and fuzzy Markov chain is used to accurately predict network security situation.

Based on pattern recognition method, it is necessary to classify all possible situations of network security situation with the help of expert knowledge and experience or machine learning, and finally determine the network security status by calculating the correlation between training samples and measured data. The methods based on pattern recognition mainly include support vector machine, neural network, clustering analysis, grey relational analysis, rough set, and so on. Xiao et al. and Wang et al. [13, 14] all use the improved SVM method to predict the network security situation. Xiao et al. [13] optimize the parameters of SVM by particle swarm optimization (PSO) algorithm and propose a PSO-SVM network security situation prediction model, which finally accurately predicts the network security situation based on small sample data. On this basis, Wang et al. [14] reduce the influence of irregular disturbance by accumulating the original sequences and proves its superiority by comparing with PSO-SVM prediction model.

At present, machine learning has become a hot spot in solving nonlinear complex problems in various research fields. Neural network, which belongs to pattern recognition methods, is one of machine learning algorithms, which has been widely used in medical, financial, management, electrical, and other fields. A large number of researchers have also predicted the network security situation by neural network.

Compared with the traditional machine learning model, the deep learning model shows great potential in the field of network security situation prediction. Tao et al. and Zhang et al. [15, 16] study the network security situation prediction model based on BP neural network. Although BP neural network for network security situation prediction has a certain effect, BP neural network algorithm deficiencies lead to a lot of limitations. The characteristics of complex scenes and dynamic changes of network security situational awareness make the occurrence of network security events have great probability and suddenness. Li and Zhao [17] propose a network security situational prediction method based on LSTM. LSTM neural network is an improvement of recurrent neural network and has strong performance in processing time series data. Kurri et al. [18] propose a network traffic prediction method based on LSTM and introduces particle filter constraint algorithm to optimize network parameters. Aiming at the problem that the slow convergence speed affects the training cost in the training process of LSTM neural network, Li et al., Zhang et al., Liu et al., and Yang et al. [19–22] propose an intelligent optimization algorithm to improve the convergence speed of LSTM neural network model. Aiming at the problems of low prediction accuracy and low efficiency in traditional neural network, Chen et al. [23] propose a new prediction method of recurrent neural network based on gated recurrent unit. This method extracts information features from the original time series data and applies them to the depth RNN model for training and verification. After iteration and optimization, the trained model can obtain the accuracy of network security prediction. Wang et al. [24] propose a prediction method based on two-layer recurrent neural network LSTM and GRU. This method combines two improved recurrent neural networks. Although the prediction accuracy is improved, the complexity of the model is increased and the training time of the model is prolonged.

In order to solve the problems existing in traditional forecasting models, this paper proposes a combined forecasting model based on EMD and ELPSO optimized BiGRU neural network (EMD-ELPSO-BiGRU). Considering that multi-attribute security index data are used as the data support, the multi-attribute network security data are fused on the basis of BiGRU neural network, the network security situation data sequence is decomposed into a series of intrinsic mode function by empirical mode decomposition, and the super-parameters and network scale of the network are determined by improved particle swarm optimization (ELPSO) algorithm, which further improves the performance of the model. This model preserves the original network security data to a great extent, maximizes the correlation between mining data, and improves the prediction accuracy.

The rest of this paper is arranged as follows: the second section introduces the related basic algorithms involved in this paper, including empirical mode decomposition, BiGRU neural network, and conventional PSO; the third section introduces the particle swarm optimization algorithm based on cooperative update of evolutionary state judgment and learning strategy proposed in this paper; the fourth section introduces the optimization of BiGRU neural network hyper-parameters based on ELPSO algorithm. The fifth section introduces the network security situation prediction model based on EMD-ELPSO-BiGRU; the sixth section discusses the experiment and results. The seventh section summarizes the work of this paper.

## 2. Correlation Basic Algorithm

### 2.1. Empirical Mode Decomposition

Empirical mode decomposition (EMD) [25] is a method to deal with nonlinear and nonstationary time-varying sequences. This method adaptively decomposes signals according to the time scale characteristics of data itself and is considered as a breakthrough in Fourier analysis and wavelet analysis based on stationary and linear assumptions. The screening process of EMD algorithm is to decompose complex time series data into a finite number of intrinsic mode function (IMF), and the IMF components obtained by decomposition contain the fluctuation information of the original data in different time scales.

For a given original time sequence sample data *x*(*t*), firstly, the local maximum and minimum values on *x*(*t*) are calculated, respectively, and the local maximum and minimum values are interpolated and fitted to obtain the upper and lower envelope network *x*_max_(*t*) and *x*_min_(*t*) sequence of the original data *x*(*t*), and then calculate the mean value of the upper and lower envelope sequence to obtain the mean value sequence *m*_1_(*t*): x(t).

(1) $$\begin{array}{c} m_{1}\left(t\right)=\frac{x_{\max}\left(t\right)+x_{\min}\left(t\right)}{2}. \end{array}$$

Subtract the mean sequence from the original sequence to get a new sequence *h*_1_^1^(*t*) with low frequency removed:

(2) $$\begin{array}{c} h_{1}^{1}\left(t\right)=x\left(t\right)-m_{1}\left(t\right). \end{array}$$

Generally speaking, *h*_1_^1^(*t*) does not meet the conditions of the eigenmode function. At this time, *h*_1_^1^(*t*) is used as the original sequence, and it is repeated *k* times until the average curve tends to zero. The judgment condition for marking *c*_1_(*t*)=*h*_1_^*k*^(*t*) and treating *c*_1_(*t*) as an IMF is

(3) $$\begin{array}{c} SD=\sum_{k=1}^{T}\frac{{\left[h_{1}^{k-1}\left(t\right)-h_{1}^{k}\left(t\right)\right]}^{2}}{{\left[h_{1}^{k-1}\left(t\right)\right]}^{2}}. \end{array}$$

Here, SD is the sieving threshold, which is generally between 0.2 and 0.3. Subtract *c*_1_(*t*) from *x*(*t*) to get the residual sequence *r*_1_(*t*)=*x*(*t*) − *c*_1_(*t*) with the highest frequency components removed. The above screening process is repeated to obtain subsequent IMF components, until *c*_*n*_(*t*) is less than the predetermined error or *r*_*n*_(*t*) is a monotonic function, and the modal decomposition process is terminated. So far, the original sequence *x*(*t*) can be represented by the n-order IMF component and the residual *r*_*n*_(*t*):

(4) $$\begin{array}{c} x\left(t\right)=\sum_{i=1}^{n}c_{i}\left(t\right)+r_{n}\left(t\right). \end{array}$$

### 2.2. BiGRU Neural Network

**Figure 1:**
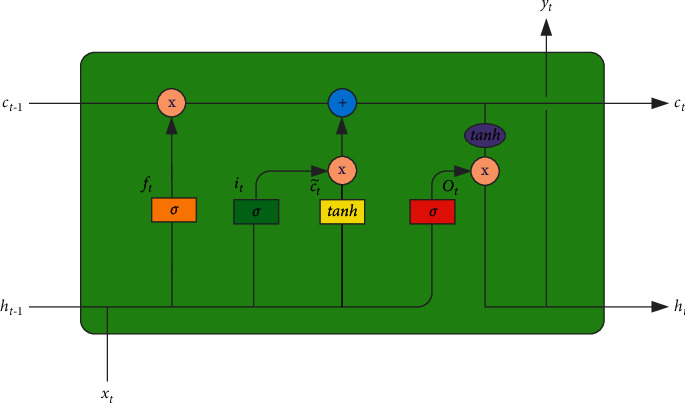
LSTM neuron structure diagram.

Bidirectional gated recurrent unit (BiGRU) is a bidirectional gated-based recurrent neural network, which is composed of forward GRU and backward GRU [26]. GRU model is a variant of long short-term memory (LSTM [27]) network. Compared with LSTM, the network structure of GRU model is simpler, but the effect is basically the same as LSTM, which greatly reduces the time required for network training. The output of the current time step of the recurrent neural network is related to the output of the previous time step, which makes the recurrent neural network have memory and is suitable for processing sequence data. However, the traditional neural network only has short-term memory, which is not effective for long-distance dependence, and has the problem of gradient explosion or gradient disappearance. LSTM solves the above problems through gating mechanism and can learn long-span dependencies. The structure of LSTM neurons is shown in Figure 1.

**Figure 2:**
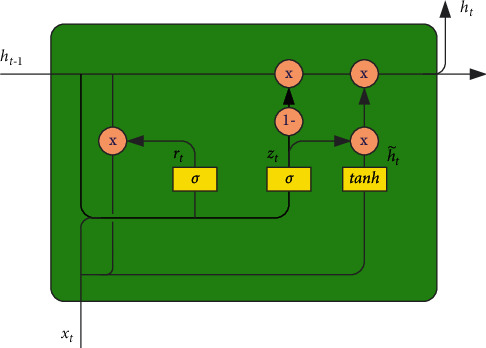
Structural diagram of GRU neurons.

GRU network combines input gate and forgetting gate in LSTM, called update gate, which greatly reduces the time required for training the network. The structure of GRU neurons is shown in Figure 2.

In the GRU network, the update gate controls how many hidden states at the historical moment and candidate states at the current time are retained in the hidden state *h*_*t*_ at the current time. The function of the reset gate is to determine the degree of dependence between the candidate state *h*_*t*_′ at the current moment and the hidden state at the previous moment.

(5) $$\begin{array}{c} z_{t}=\sigma \left(w_{z}x_{t}+u_{z}h_{t-1}+b_{z}\right), \end{array}$$

(6) $$\begin{array}{c} r_{t}=\sigma \left(w_{r}x_{t}+u_{r}h_{t-1}+b_{r}\right), \end{array}$$

(7) $$\begin{array}{c} h_{t}^{\prime }=\tanh\left(w_{c}x_{t}+u_{c}\left(r_{t}⊙h_{t-1}\right)+b_{c}\right), \end{array}$$

(8) $$\begin{array}{c} h_{t}=z_{t}⊙h_{t-1}+\left(1-z_{t}\right)⊙h_{t}^{\prime }, \end{array}$$

(9) $$\begin{array}{c} y_{t}=\sigma \left(W_{0}\cdot h_{t}\right), \end{array}$$

*x*_*t*_ is the input at the current moment, *h*_*t*−1_ is the hidden state at the previous moment, *h*_*t*_′ is the candidate state at the current moment, *h*_*t*_ is the hidden state at the current moment, and *y*_*t*_ is the output at the current moment. Formula (8) is the calculation formula of the update gate, and formula (9) is the calculation formula of the reset gate.

**Figure 3:**
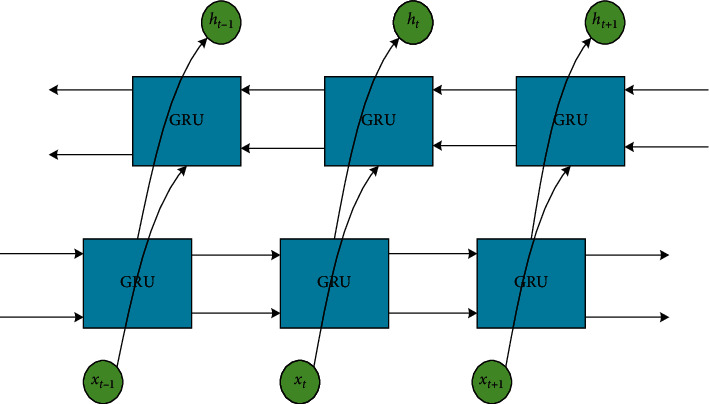
BiGRU network structure diagram.

In the GRU network, information can only be transmitted in one direction, but in practice, each input data may have a dependency on the input data before and after it. Using the BiGRU network through trainment data network in two directions makes the model more effective. The structure of BiGRU network is shown in Figure 3.

### 2.3. Conventional PSO

Particle swarm optimization (PSO) is an intelligent search algorithm that simulates the social behavior of bird groups [28] and searches the solution of the problem cooperatively through information sharing among individuals in the group. The specific mathematical description of the algorithm is assuming that the dimension of the target search space is *D*, the particle population size is *N*, *X*_*i*_=(*x*_*i*1_, *x*_*i*2_,…, *x*_*iD*_) represents the position of the *i*-th particle in the D-dimensional search space, and *V*_*i*_=(*v*_*i*1_, *v*_*i*2_,…, *v*_*iD*_) represents the velocity of the i-th particle, where *i*=(1,2,…*N*). *p*_*best*_ represents the optimal position experienced by the *i*-th particle itself and *g*_*best*_ represents the optimal position experienced by the entire group. In the whole evolution process of the algorithm, each particle updates its own speed and position by continuously updating *p*_*best*_ and *g*_*best*_, so as to find the best position of the particle when it reaches the optimal fitness value, which is the solution of the problem to be optimized. The particle velocity and position update formulas are

(10) $$\begin{array}{c} V_{i}^{t+1}=\omega V_{i}^{t}+c_{1}r_{1}\left(p_{best}-x_{i}^{t}\right)+c_{2}r_{2}\left(g_{best}-x_{i}^{t}\right), \end{array}$$

(11) $$\begin{array}{c} X_{i}^{t+1}=X_{i}^{t}+V_{i}^{t+1}. \end{array}$$

Among them, *ω* is the inertia weight; *c*_1_ and *c*_2_ are learning factors, and usually the value is 2; *r*_1_ and *r*_2_ are random numbers distributed in [0, 1]; *t* is the current iteration number of the particle.

## 3. Particle Swarm Optimization Algorithm Based on Cooperative Update of Evolutionary State Judgment and Learning Strategy

PSO algorithm has the advantages of simple structure, few control parameters, outstanding global optimization ability, etc. It also has the characteristics of fast calculation speed, few parameters, and convenient implementation. However, the algorithm has some problems in the search process, such as premature convergence or falling into local optimum, which is mainly due to the loss of population diversity in the optimization process [29]. Keeping population diversity is an important measure to enhance the global search ability and avoid premature phenomenon. Therefore, in this paper, the learning strategy updating mechanism based on evolutionary state information decision is adopted in the iterative process of particle swarm optimization algorithm, and ELPSO is proposed.

Different from the traditional particle swarm optimization algorithm, ELPSO algorithm uses the information of population evolution to choose the appropriate learning strategy. When the evolutionary state is greater than the fixed threshold, the decision algorithm is in the convergence stage, and the full information learning strategy is adopted to update the speed and position of the information of the particles in the better neighborhood to speed up the convergence speed of the algorithm; when the evolution state is less than a fixed threshold, the decision algorithm is in the stage of jumping out of local optimum. The algorithm adopts local information learning strategy and updates the speed and position of local optimum and best neighborhood particles according to the information, so as to maintain the diversity of population and make the algorithm difficult to fall into local optimum.

### 3.1. Evolutionary State Analysis

In the iterative process of particle swarm optimization, the reduction of population diversity is the main reason why particle swarm optimization falls into local optimum. In view of this characteristic and the linear relationship between iteration times and population diversity, this paper defines the evolution factor *E*, and its calculation formula is

(12) $$\begin{array}{c} x_{\text{mean}}^{d}=\frac{1}{N}\sum_{i=1}^{N}x_{i}^{d}, \end{array}$$

(13) $$\begin{array}{c} \text{div}=\frac{1}{N}\sum_{i=1}^{N}\sqrt{\overset{D}{\underset{d=1}{\sum }}\left(x_{i}^{d}-x_{\text{mean}}^{d}\right)\left(x_{i}^{d}-x_{\text{mean}}^{d}\right)}, \end{array}$$

(14) $$\begin{array}{c} E=e^{-\text{div}/{\text{div}}_{\max}/k}\left(1-\frac{t}{T}\right)\in \left[0,1\right]. \end{array}$$

In the formula, *x*_mean_^*d*^ represents the average position between particles at the same latitude; *N* represents the total number of populations; *D* represents the particle dimension; *k* is used to adjust the sensitivity of the exponential function and is matched according to the initialization state of the population and the degree of population diversity; *t* is the current number of iterations; *T* is the maximum number of iterations; and div and div_max_ represent the values of the current population diversity and the maximum population diversity, respectively, when the two are equal, *E*=1.

### 3.2. Neighborhood Selection Strategy

In the iterative process, according to the coding characteristics of particle swarm optimization, the Hamming distance between each particle and other particles is calculated, and they are sorted. According to the sorting results, the neighbors of a given particle with a specified number are obtained.

(15) $$\begin{array}{c} D_{ij}=H\left(x_{i},x_{j}\right), \end{array}$$

(16) $$\begin{array}{c} S=\text{sort}\left(D\right), \end{array}$$

(17) $$\begin{array}{c} \text{Neighbors}=S\left(1:T\right). \end{array}$$

In the formula, *D*_*ij*_ represents the Hamming distance between the *i*-th particle and the *j*-th particle in the population; *H* is the function to calculate the Hamming distance; *S* is the set of sorting results; *Neighbors* represents the current neighborhood particle set; and *T* is the specified number of neighbors.

### 3.3. Full Information Learning Strategies

In order to improve the particle optimization problem, ELPSO algorithm adopts full information learning strategy to ensure the optimization ability and convergence performance. In the iterative process of the ELPSO algorithm, particle *i* obtains information from neighboring particles with better fitness value and at the same time avoids the influence of bad neighboring particles. The neighboring particles with better fitness value have greater influence on particle *i*. Based on the above ideas, the ELPSO algorithm adopts a full information learning strategy, and its speed and position update expressions are

(18) $$\begin{array}{c} V_{i}^{d}=X\left(V_{i}^{d}+\frac{\varphi }{k_{i}}\sum_{m=1}^{k_{i}}r_{m}\cdot \frac{f\left(p_{im}\right)}{{\text{sum}}_{i}}\cdot \left(p_{{}_{im}}^{d}-x_{i}^{d}\right)\right), \end{array}$$

(19) $$\begin{array}{c} {\text{sum}}_{i}=\sum_{m=1}^{k_{i}}f\left(p_{im}\right), \end{array}$$

(20) $$\begin{array}{c} S\left(V_{i}^{d}\right)=1-\text{sqrt}\left({\left(1-V_{i}^{d}\right)}^{2}\right), \end{array}$$

According to the relevant literature, using the convergence coefficient *X* and the acceleration coefficient *φ* to adjust the particle velocity, the algorithm performance is better, where *X*=0.729, *φ*=4.1; *k*_*i*_ is the number of particles in the better neighborhood of particle *i*; *i*_*m*_ is the *m*-th better neighborhood particle of particle *i*; *p*_*im*_ is the position of the particle *i*_*m*_; *f*(*p*_*im*_) is the fitness value of the particle; sum(*i*) is the sum of the fitness values of the particles in the better neighborhood; and *r*_*m*_ represents a number uniformly distributed between [0,1]. Equation (20) is the particle position update formula. *V*_*i*_^*d*^ is the particle velocity value, and *S*(*V*_*i*_^*d*^) is the probability value of the velocity mapping. If the probability value is greater than the random number rand, the particle position vector takes its own complement; otherwise it remains unchanged.

### 3.4. Local Information Learning Strategies

In particle swarm optimization, particle *i* updates speed and position according to the information of local optimal and optimal neighbor particles, is less affected by other particles, and can move more freely in the search space, which is conducive to maintaining population diversity. ELPSO adopts the local information learning strategy, and its speed and position update expressions are

(21) $$\begin{array}{c} V_{i}^{d}=X\left[V_{i}^{d}+\frac{\varphi }{2}r_{1}\left(p_{i}^{d}-x_{i}^{d}\right)+\frac{\varphi }{2}r_{2}\left(p_{i_{nb}}^{d}-x_{i}^{d}\right)\right]. \end{array}$$

In the formula, *p*_*i*_^*d*^ is the local optimal position of the particle *i*; *p*_*i*_*nb*__^*d*^ is the optimal position of the neighborhood of particle *i*; and *r*_1_ and *r*_2_ represent the numbers evenly distributed between [0, 1].

### 3.5. ELPSO Algorithm Flow

The ideal particle swarm optimization algorithm should not fall into local optimum while ensuring fast convergence speed, which is difficult to achieve by using a single learning strategy. Therefore, ELPSO algorithm adopts different learning strategies to solve complex optimization problems in different evolutionary states. Aiming at the problem that particle swarm optimization is premature and easy to fall into local optimum, the iterative process of particle swarm optimization is divided into two stages: jumping out of local optimum and converging. At the same time, the evolution state is divided. If the evolution factor *E* < 0.7, it is judged that the algorithm is in the stage of jumping out of local optimum, which shows that the population diversity is poor. Local information learning strategy should be selected to ensure that particles can move more freely in the search space to maintain the population diversity; if the evolutionary factor *E* > 0.7 or *E* = 0.7, the decision algorithm is in the convergence stage, which shows that the population diversity is good. All-information learning strategy should be selected to ensure that particles get information from neighborhood particles with better fitness value to accelerate convergence. The specific steps of ELPSO are

  Step 1. Population initialization. Set particle population size, learning rate factor, iteration times, and search space dimension.
  Step 2. Evolution state determination. Calculate the evolution factor *E*; if *E* < 0.7, it is judged that the algorithm is in the stage of jumping out of local optimum; if *E* ≥ 0.7, the decision algorithm is in convergence stage.
  Step 3. Particle velocity update. If the algorithm is in the stage of jumping out of local optimum, the particle velocity is updated by formula (21); if the algorithm is in the convergence stage, the particle velocity is updated by equations (18) and (19).
  Step 4. Update particle position. The particle position is updated by equation (20).
  Step 5. Repeat steps 2 to 5 until the termination condition is met.
  Step 6. satisfies the termination condition (reaching the maximum iteration times), outputs the optimal value, and obtains the corresponding objective function value, and the algorithm ends.

**Figure 4:**
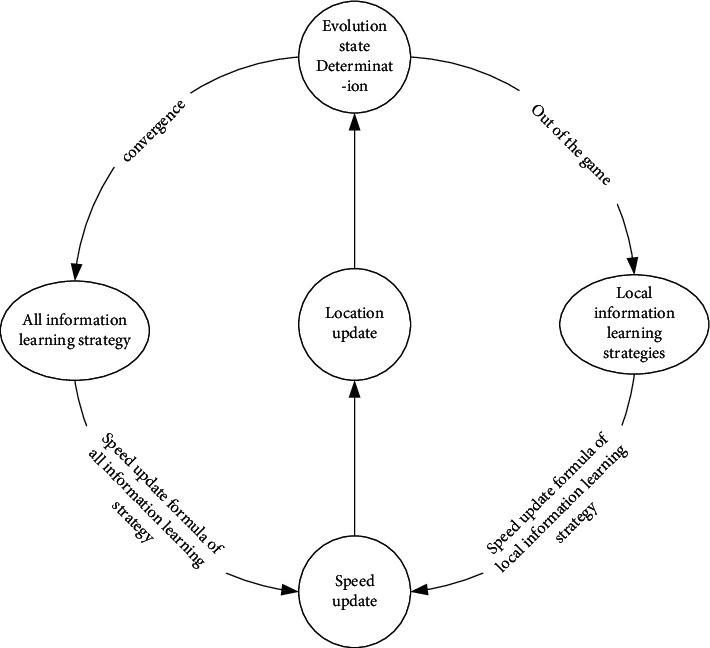
Algorithm optimization mechanism.

In ELPSO algorithm, evolutionary state judgment is the key to balance convergence and jump out of local optimum. The optimization mechanism of particle swarm optimization algorithm in which evolutionary state judgment and learning strategy are updated cooperatively is shown in Figure 4.

## 4. Optimization of Hyper-Parameters of BiGRU Neural Network Based on ELPSO Algorithm

When ELPSO algorithm is used to optimize BiGRU network, this paper uses supervised learning to train the model in the training stage of BiGRU network and takes the mean square error function as the loss function of the model.

Its mathematical definition is as follows:

(22) $$\begin{array}{c} \text{MSE}=\frac{1}{N}\sum_{i=1}^{N}{\left(y_{i}-{\hat{y}}_{i}\right)}^{2}. \end{array}$$

where *N* is the number of training samples, *y*_*i*_ is the actual value, and ${\hat{y}}_{i}$ is the model prediction value.

The training data of BiGRU neural network involve the setting of several super-parameters: the number of neurons *m*, the time step *T*, and the batch size. The number of neurons determines the fitting degree of neural network, and the time step and batch size directly affect the training results of the model. In practical application, different super-parameter settings corresponding to different data sets will affect the prediction accuracy. In this paper, ELPSO is used to optimize these super-parameters, and according to the input data, the neural network structure and training mode are adaptively optimized to obtain the optimal combination of model parameters. The specific steps are as follows:

  Step 1: initializes the parameters of the algorithm, and determine the population size, iteration times, inertia weight, and the change interval of the learning factor.
  Step 2: randomly generates a three-dimensional population particle (*M*, *T*, batch size) and initializes the position and velocity of the particle, and the dimension of the particle is the parameter to be optimized.
  Step 3: takes formula (22) as the fitness function of the particle. The smaller the fitness function, the smaller the loss function of the model, and the better the parameter combination obtained by the particle.
  Step 4: updates the velocity and position of particles.
  Step 5: stops when the number of iterations is reached or the fitness function of particles tends to be stable, and the particles at the best position of the population are the optimal parameter combination obtained this time; otherwise, turn to Step 4 to continue iteration.

**Figure 5:**
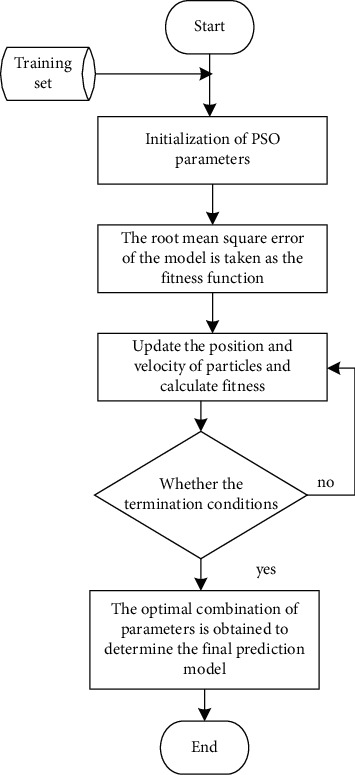
Flowchart of ELPSO optimizing BiGRU.

The flowchart of using ELPSO algorithm to solve the optimal parameter combination of BiGRU model is shown in Figure 5.

## 5. Network Security Situation Prediction Model Based on EMD-ELPSO-BiGRU

**Figure 6:**
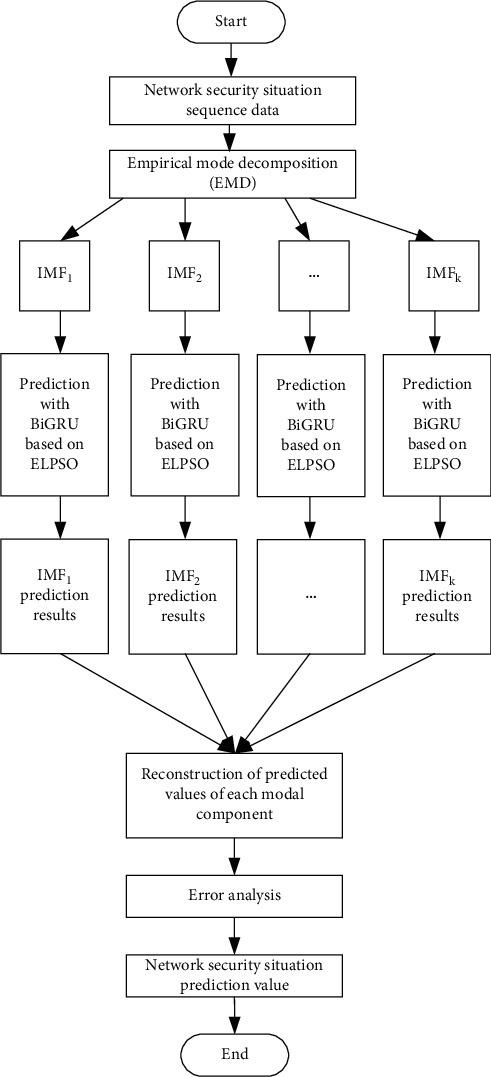
Flowchart of network security situation prediction based on EMD-ELPSO-BiGRU.

In order to analyze the characteristics of network security situation change in detail, this paper proposes a combined prediction model (EMD-ELPSO-BiGRU) based on empirical mode decomposition and improved particle swarm optimization (ELPSO) to optimize BiGRU neural network. Firstly, the network security situation sequence is stabilized by variational empirical mode decomposition, which is decomposed into a series of different modal components to reduce the complexity of the network security situation sequence; then, BiGRU neural network optimized based on ELPSO algorithm is used to predict each modal component; finally, the prediction results of each modal component of the network security situation sequence are superimposed to obtain the network security situation prediction value. The network security situation prediction process is shown in Figure 6.

## 6. Experiences and Discussion

### 6.1. Performance Evaluation of ELPSO Algorithm

#### 6.1.1. Benchmark Function

In order to test the effectiveness of ELPSO proposed in this paper, conventional particle swarm optimization (PSO) [30], improved particle swarm optimization (MPSO) [31], quantum particle swarm optimization (QPSO) [32], IAP-PSO [33], EIW-PSO [34], CLPSO [35], and SRPSO [36] are selected for comparative experiments on 12 benchmark functions. The mathematical expressions for the 12 test functions are shown below [37].

(1) Sphere function
  (23) $$\begin{array}{c} f_{1}\left(x\right)=\sum_{i=1}^{N}x_{i}^{2}. \end{array}$$
(2) Schwefel function
  (24) $$\begin{array}{c} f_{2}\left(x\right)=\max\left\{\left|x_{i}\right|,1\leq i\leq n\right\}. \end{array}$$
(3) Schwefel function
  (25) $$\begin{array}{c} f_{3}\left(x\right)=\overset{n}{\underset{i=1}{\sum }}\left|x_{i}\right|+\prod_{i=1}^{n}\left|x_{i}\right|. \end{array}$$
(4) Step function
  (26) $$\begin{array}{c} f_{4}\left(x\right)=\sum_{i=1}^{n}{\left(x_{i}+0.5\right)}^{2}. \end{array}$$
(5) Schaffer function
  (27) $$\begin{array}{c} f_{5}\left(x\right)=\sum_{i=1}^{n}{\left(x_{i}^{2}+x_{i+1}^{2}\right)}^{0.25}\left[\sin^{2}\left(50{\left(x_{i}^{2}+x_{i+1}^{2}\right)}^{0.1}\right)+1.0\right]. \end{array}$$
(6) Rastrigin function
  (28) $$\begin{array}{c} f_{6}\left(x\right)=\sum_{i=1}^{N}\left(x_{i}^{2}-100\cos\left(2\pi x_{i}\right)+10\right). \end{array}$$
(7) Griewank function
  (29) $$\begin{array}{c} f_{7}\left(x\right)=\frac{1}{4000}\sum_{i=1}^{n}x_{i}^{2}-\prod_{i=1}^{n}\cos\left(\frac{x_{i}}{\sqrt{i}}\right)+1. \end{array}$$
(8) Ackley function
  (30) $$\begin{array}{c} f_{8}\left(x\right)=-20\exp\left(-0.2\times \sqrt{\frac{1}{n}\sum_{i=1}^{n}x_{i}^{2}}\right)-\exp\left(\frac{1}{n}\sum_{i=1}^{n}\cos\left(2\pi x_{i}\right)\right)+20+e. \end{array}$$
(9) Schaffer function
  (31) $$\begin{array}{c} f_{9}\left(x\right)=0.5+\frac{\left(\sin^{2}\sqrt{x_{1}^{2}+x_{2}^{2}}-0.5\right)}{{\left(1+0.001\left(x_{1}^{2}+x_{2}^{2}\right)\right)}^{2}}. \end{array}$$
(10) Branin function
  (32) $$\begin{array}{c} f_{10}\left(x\right)={\left(x_{2}-\frac{5.1}{4\pi }x_{1}^{2}+\frac{5}{\pi }x_{1}-6\right)}^{2}+10\left(1-\frac{1}{8\pi }\right)\cos x_{1}+10. \end{array}$$
(11) Six-hump camel back function
  (33) $$\begin{array}{c} f_{11}\left(x\right)=4x_{1}^{2}-2.1x_{1}^{4}+\frac{x_{1}^{6}}{3}+x_{1}x_{2}-4x_{2}^{2}+4x_{2}^{4}. \end{array}$$
(12) Goldstein price function
  (34) $$\begin{array}{c} f_{12}\left(x\right)=\left[1+{\left(x_{1}+x_{2}+1\right)}^{2}\left(19-14x_{1}+3x_{1}^{2}-14x_{2}+6x_{1}x_{2}+3x_{2}^{2}\right)\right]\\ \times \left[30+{\left(2x_{1}-3x_{2}\right)}^{2}\left(18-32x_{1}+12x_{1}^{2}+48x_{2}-36x_{1}x_{2}+27x_{2}^{2}\right)\right]. \end{array}$$

#### 6.1.2. Analysis of Simulation Results

In the experiment, different PSO algorithms set the same population size *N*=40, the maximum number of iterations is *T*_max_=500, the learning factor *c*_1_=*c*_2_=2, and other parameter settings are consistent with the original literature; in the ELPSO algorithm, *w*_max_=0.9, *w*_min_=0.4, *σ*=0.1.

In order to test the performance of the algorithm, the experiments were divided into three groups, the dimensions of the algorithm were set to 10, 30, and 50, and the four algorithms were run independently for 50 times. The mean value (MEAN) of the test results of each algorithm is shown in Tables 1–3.

From the comparison results of Tables 1–3, it can be obtained that on the 12 test functions, compared with other algorithms, the ELPSO algorithm has further improved the optimization effect of the test function and has better stability; whether in low or high dimensions, the ELPSO algorithm can find better results in unimodal, multimodal, and combined functions.

#### 6.1.3. *T* Test and Friedman Test

In order to further clarify whether there are significant differences between algorithms, this paper introduces *T* test [38] and Friedman test [39] to test the performance of 8 algorithms on 12 test functions from a statistical point of view. The experimental results are shown in Table 4. The *T* test results show that the performance difference between ELPSO algorithm and other algorithms is obvious; compared with PSO, ELPSO has better performance in 9 test functions, and there is no difference in 3 test functions. Compared with MPSO, ELPSO has 7 better functions, 3 no difference, and 1 worse; compared with QPSO, four functions of ELPSO are better, six have no difference, and two are worse; compared with IAP-PSO, ELPSO has better 8 functions and no difference in 4 functions. Compared with EIW-PSO, ELPSO has 7 better functions, 4 no difference, and 1 worse; compared with CLPSO, ELPSO has 3 better functions, 7 no difference, and 2 worse functions; compared with SRPSO, ELPSO has better 7 functions and no difference in 5 functions. The Friedman test results of 8 algorithms show that the rank mean of ELPSO algorithm is the smallest, and the performance of ELPSO algorithm is the best among the 8 algorithms. Combining the two test results, we can see that the performance of ELPSO algorithm is better than other algorithms, where “+” indicates that ELPSO algorithm is superior to other algorithms, “=” indicates that there is no obvious difference between algorithms, “−” indicates that ELPSO algorithm is inferior to other algorithms, and *w*/*t*/*l* indicates the statistical number of these three comparison results, respectively.

#### 6.1.4. Wilcoxon Rank Test

Referring to the data statistics and analysis methods in reference [40], Wilcoxon rank test with significance level of 0.05 is used to judge the performance of the algorithm. Among them, “+,” “−,” and “≈,” respectively, indicate that the results of ELPSO algorithm are better than, worse than, and equivalent to the test results of corresponding algorithms.

From the Wilcoxon results in Table 5, when *α*=0.05, the ELPSO algorithm has obtained obvious advantages compared with the comparison algorithm in the test function. It can be seen that compared with other algorithms, the ELPSO algorithm has outstanding advantages in solving high-dimensional problems.

#### 6.1.5. Average Number of Iterations at Specified Precision

In order to comprehensively analyze the performance of the algorithm, this section gives 8 algorithms to test 12 benchmark functions under the specified precision of 10^−10^, the dimension is 30, and the average number of iterations for each algorithm runs independently for 50 times. The results are shown in Table 6.

From the experimental results in Table 6, it can be seen that the PSO algorithm only achieves the specified accuracy on 3 test functions, and the MPSO algorithm achieves the specified accuracy on 10 functions. However, QPSO, IAP-PSO, EIW-PSO, CLPSO, SRPSO, and ELPSO achieve the specified accuracy in all test functions. And compared with other algorithms, the ELPSO algorithm can achieve the specified accuracy with the least number of iterations, and the average number of iterations is between 11 and 34. This shows that the convergence speed of the ELPSO algorithm has obvious advantages and high optimization performance, which further shows that the ELPSO algorithm has the characteristics of fast convergence speed.

### 6.2. Simulation Analysis of Network Security Situation Prediction

#### 6.2.1. Selection of Network Security Situation Data

**Figure 7:**
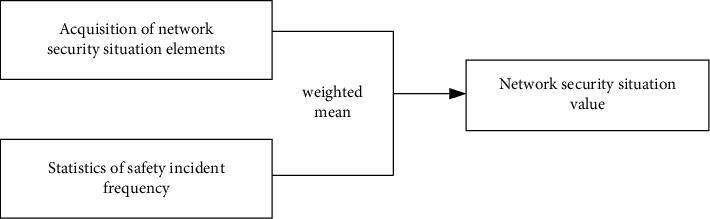
Quantitative model of network security situation assessment.

In this paper, the weekly data of security situation released by the National Internet Emergency Center are used as the experimental basis [41]. The National Internet Emergency Center is a network security technology coordination organization in Chinese mainland, which mainly processes the national security incidents statistically, evaluates the network security status, and publishes security information on a weekly, monthly, and annual basis. The dynamic weekly report mainly evaluates the basic situation of network security with five security indicators, including the number of hosts infected with network viruses in China, the total number of tampered websites in China, the total number of backdoor websites implanted in China, the number of phishing pages of domestic websites, and the number of new information security vulnerabilities. In this paper, 120 safety data from the 31st issue of 2017 to the 45th issue of 2019 are selected as experimental basis to verify the superiority of this method. The evaluation method of reference [42] is cited to quantify the original data, and the network security situation value of 120 weeks is obtained. The specific quantification model is shown in Figure 7.

#### 6.2.2. Experimental Data and Its Preprocessing

**Figure 8:**
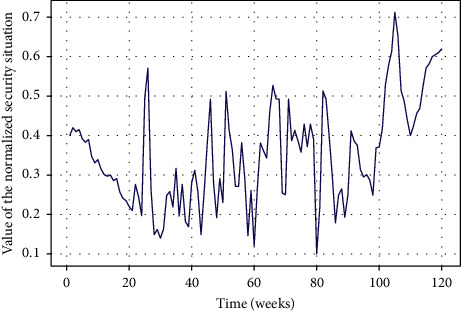
Normalized network security situation time series.

In this paper, the data of the first 101 weeks are selected as the training set and the data of the last 18 weeks as the test set according to the time sequence. The time window is set as the time step of the recurrent neural network, and the prediction time is one week. Because of the complexity and randomness of the network environment and the great difference of the dimensions of situation values, the activation function of the neural network used in this paper is extremely sensitive to whether the input data are within [−1, 1]. Therefore, standardizing the data can accelerate the convergence speed and improve the prediction accuracy of the neural network. The input data are processed by data normalization, and the specific calculation formula is as follows [43].

(35) $$\begin{array}{c} x^{\prime }=\frac{x-\min\left(x\right)}{\max\left(x\right)-\min\left(x\right)}, \end{array}$$

where *x* and *x*′ are the data before processing, and min(*x*) and max(*x*) are the minimum and maximum values in the data set. Therefore, the normalized network security situation value is shown in Figure 8.

#### 6.2.3. Model Metrics and Evaluation Indicators

In this paper, two measurement methods are selected to evaluate the proposed prediction model: mean absolute error and root mean square error. The specific formula is defined as follows:

(36) $$\begin{array}{c} \text{MAE}=\frac{1}{N}\sum_{i=1}^{\text{N}}\left|y_{i}-{\hat{y}}_{i}\right|, \end{array}$$

(37) $$\begin{array}{c} \text{RMSE}=\sqrt{\frac{1}{N}\sum_{i=1}^{\text{N}}{\left(y_{i}-{\hat{y}}_{i}\right)}^{2}}, \end{array}$$

where *N* is the number of training samples, *y*_*i*_ is the actual value, and ${\hat{y}}_{i}$ is the predicted value.

After preprocessing the network security situation data, the ELPSO algorithm can be used to obtain the optimal combination of model parameters. Initialize ELPSO: the population size of the PSO algorithm is 5, the evolution times are 40, and the dimension of each particle is 3, which, respectively, represent the parameters to be optimized—the number of encoder neurons, the number of prediction network neurons, the time step *T*, and the batch size. For simplicity, the number of encoder neurons is set to be equal to the number of prediction network neurons. The maximum value of learning factors *c*_1_ and *c*_2_ is 2.5, the minimum value is 0.5, and the weight factor *w* is 0.8.

#### 6.2.4. Optimal Parameter Selection of the Model

**Figure 9:**
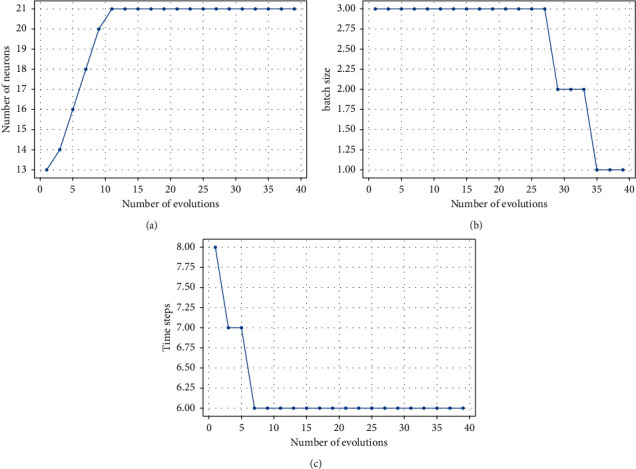
ELPSO optimizations—the parameters of BiGRU. (a) Number of neurons. (b) Batch size. (c) Time steps.

Figure 9 shows the training results of ELPSO algorithm optimizing BiGRU neural network. The number of neurons, time step size, and batch size gradually converge to the optimal value with the update of the algorithm. As can be seen from Figure 9, the number of neurons finally converges to 21, the batch size of model training data is 1, and the optimal time step is 6. So far, the best super-parameters are obtained to modify the model structure of BiGRU neural network and obtain the best parameter combination.

#### 6.2.5. Analysis of Simulation Experiment Results

In order to evaluate the performance of the proposed model in network security situation prediction, comparative experiments are carried out with traditional machine learning methods and deep learning methods, including BP [44], LSTM [45], BiGRU [46], and ELPSO-BiGRU models. The experimental environment of this paper is Windows 10 operating system, and Keras deep learning framework is used for model training and testing in Python3.7 environment, hardware configuration: 64-bit operating system with Inter (R) Core (TM) i5-8500 CPU 3.00 GHZ processor.

**Figure 10:**
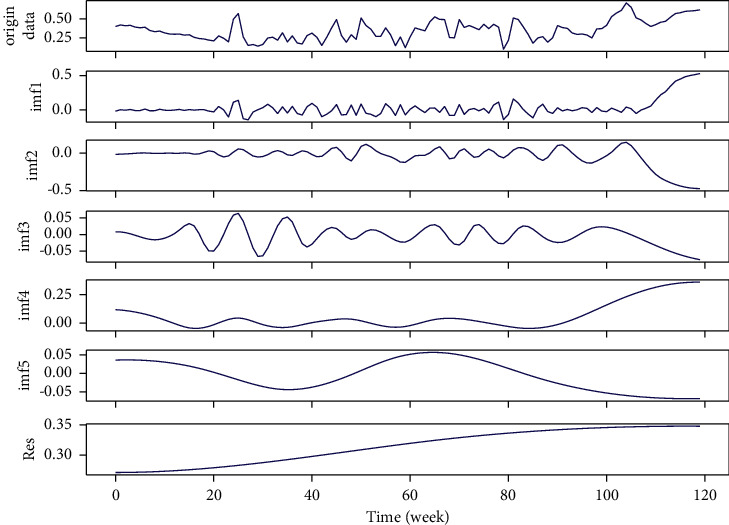
EMD results.

*(1) EMD Decomposition of Experimental Data*. Firstly, EMD is carried out on the network security situation data sequence, the number of modal components is adaptively obtained in the recursive process, and five intrinsic modal functions and a residual component *R* are obtained, as shown in Figure 10. According to the characteristics of modal components after decomposition of network security situation data sequence, it is generally believed that high frequency components reflect the random influence of network security situation; some lower frequency components also have strong sinusoidal fluctuation characteristics, which can be considered as periodic components of network security situation data series; the low frequency part is the trend item of network security situation, which can clearly show the long-term trend of network security.

*(2) Comparison of Prediction Accuracy*. In order to evaluate the prediction ability of each model as a whole, the final two errors of different models are calculated, and the results are shown in Table 7. In order to increase the fairness of comparison, this paper carries out many experiments on all prediction models to take the average value. According to the average absolute error and root mean square error selected in this paper to measure the accuracy of the prediction results, the two evaluation indicators, respectively, represent the deviation between the predicted value and the real value and the fitting accuracy. The smaller the value, the better the prediction effect. As can be seen from Table 7, the EMD-ELPSO-BiGRU model has greater advantages than other models in overall error. Compared with the ELPSO-BiGRU model, the error is reduced by 60.9%, compared with the BiGRU model, the error is reduced by 78.3%, and compared with the prediction model of the BP neural network, the error is reduced by 97.8%, indicating that the EMD-ELPSO-BiGRU model is effective for the prediction of network security situation data.

The results in Table 8 can further prove that the EMD-ELPSO-BiGRU model can obtain good prediction results at most time points. Table 8 shows the absolute errors of different prediction models at each time point during prediction. It can be seen that the absolute errors of this method are all controlled within 0.004 and most of the errors are one order of magnitude lower than 0.004, with higher prediction accuracy than other models.

**Figure 11:**
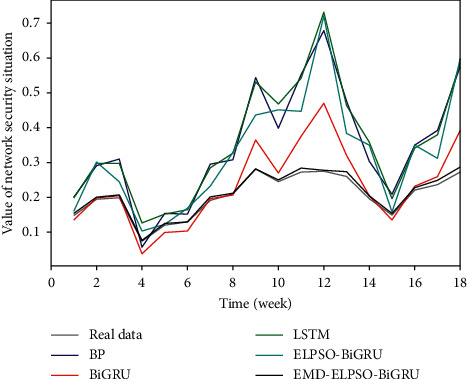
Comparison of situation prediction of different prediction models.

Figure 11 shows the comparison of prediction accuracy between EMD-ELPSO-BiGRU and basic prediction model models such as BiGRU, LSTM, and BP, and BiGRU neural network optimized based on ELPSO algorithm. It can be seen intuitively from the figure that all prediction models have a certain prediction ability, but the prediction value of the EMD-ELPSO-BiGRU model has the highest fitting degree with the real value and almost coincides with the real value at each prediction point.

**Figure 12:**
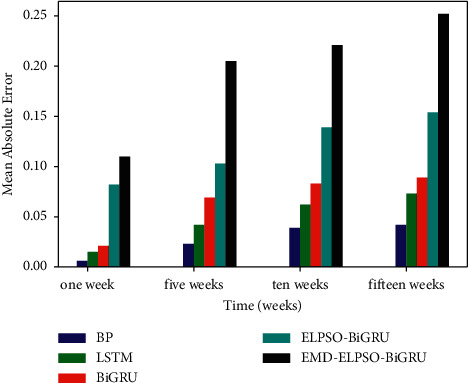
Comparison of mean absolute error under a different forecast time.

*(3) Prediction Time Comparison*. The evaluation criteria of time series prediction not only depend on the accuracy of prediction, but also depend on the accuracy under different prediction durations. In this paper, the prediction accuracy of different prediction models under different prediction duration is compared, and the results are shown in Figure 12. It can be seen that all models have the smallest error in single-step prediction. Under the same prediction time, EMD-ELPSO-BiGRU model has better prediction ability. With the increase of prediction time, the prediction error gradually increases and then changes stably, and the model has certain robustness.

**Figure 13:**
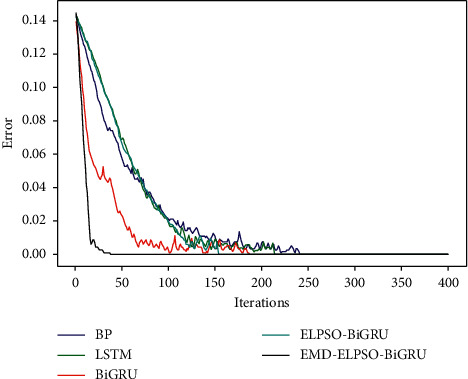
Curve of training error changing with the number of iterations.

*(4) Convergence Analysis*. In the previous section, the complexity of training different models once was compared.  Figure 13 shows the change of training error of the model with the number of iterations. It can be observed from the figure that the method in this paper has significant advantages in convergence speed and convergence accuracy, which shows that the model can learn data well.

## 7. Convention

In this paper, a combined prediction model of network security situation based on the EMD-ELPSO-BiGRU model is established for network security situation data series. Firstly, the network security situation data are decomposed by the EMD algorithm, and the BiGRU neural network based on ELPSO optimization is used to predict. In the experiment, firstly, the paper compares the proposed ELPSO algorithm with PSO and QPSO to optimize the benchmark function; then, the EMD-ELPSO-BiGRU, BP, LSTM, BiGRU, and ELPSO-BiGRU models are used to predict the network security situation, and the following conclusions can be drawn:

1. ELPSO algorithm adopts full information learning strategy in the convergence stage based on evolutionary state judgment, which has faster convergence speed than other algorithms; in the stage of jumping out of local optimum based on evolutionary state judgment, local information learning strategy is adopted to effectively avoid the algorithm falling into local optimum by maintaining population diversity.
2. Empirical mode decomposition decomposes the network security situation sequence thoroughly, which can reduce the nonstationarity of the data sequence. When the data after empirical mode decomposition are predicted by neural network, the network has higher prediction accuracy and generalization ability.
3. Compared with traditional BP neural network, LSTM, BiGRU, and ELPSO-GRU, EMD-ELPSO-BiGRU model improves the prediction accuracy of network security situation prediction.
4. The EMD-ELPSO-BiGRU prediction model proposed in this paper is universal, which is not only suitable for network security situation prediction, but also suitable for ship motion posture prediction and stock price prediction.

In the follow-up research, we will focus on the combination of deep learning models such as BiGRU and swarm intelligence algorithms such as PSO and GA to further enhance the effect of deep learning models such as LSTM in practical application.

## Figures and Tables

**Table 1:** Test results of 8 algorithms (*D* = 10).

Benchmark function	PSO	MPSO	QPSO	IAP-PSO	EIW-PSO	CLPSO	SRPSO	ELPSO
*f*_1_	2.36*E* + 02	1.30*E* + 03	7.76*E* + 02	2.70*E* + 02	3.56*E* + 03	4.02*E* + 02	8.88*E* + 02	0.00*E* + 00
*f*_2_	3.04*E* + 05	2.54*E* + 06	6.31*E* + 06	1.28*E* + 06	1.16*E* + 07	5.00*E* + 06	2.57*E* + 06	1.25*E* + 05
*f*_3_	1.08*E* + 03	1.92*E* + 03	1.57*E* + 03	2.81*E* + 02	6.42*E* + 02	2.01*E* + 03	1.20*E* + 03	2.24*E* + 02
*f*_4_	6.01*E* − 01	1.89*E* + 00	1.35*E* + 00	2.45*E* − 01	4.14*E* + 00	1.86*E* + 00	1.16*E* + 00	2.08*E* − 01
*f*_5_	9.28*E* + 01	1.49*E* + 02	1.17*E* + 02	1.51*E* + 01	6.72*E* + 00	5.25*E* + 01	3.18*E* + 01	3.00*E* + 00
*f*_6_	8.97*E* + 01	1.57*E* + 02	1.21*E* + 02	1.45*E* + 01	2.03*E* + 01	6.13*E* + 01	4.56*E* + 01	1.42*E* + 01
*f*_7_	7.96*E* + 00	2.08*E* + 01	1.26*E* + 01	2.63*E* + 00	9.06*E* − 00	2.99*E* + 02	5.24*E* + 01	4.72*E* − 01
*f*_8_	3.61*E* + 02	5.57*E* + 02	4.72*E* + 02	5.28*E* + 01	8.11*E* + 01	5.19*E* + 02	3.99*E* + 02	2.00*E* + 02
*f*_9_	1.37*E* + 03	1.91*E* + 03	1.65*E* + 03	3.15*E* + 02	1.90*E* + 03	7.88*E* + 02	3.06*E* + 02	1.60*E* + 02
*f*_10_	1.22*E* + 03	2.05*E* + 03	1.55*E* + 03	9.21*E* + 02	1.91*E* + 03	1.38*E* + 03	2.76*E* + 02	2.03*E* + 02
*f*_11_	5.96*E* + 02	1.99*E* + 03	1.32*E* + 03	3.55*E* + 02	4.49*E* + 02	1.69*E* + 03	1.04*E* + 03	2.84*E* + 02
*f*_12_	4.13*E* + 02	1.77*E* + 03	8.19*E* + 02	3.53*E* + 02	3.87*E* + 02	2.33*E* + 03	1.03*E* + 03	2.03*E* + 02

**Table 2:** Test results of 8 algorithms (*D* = 30).

Benchmark function	PSO	MPSO	QPSO	IAP-PSO	EIW-PSO	CLPSO	SRPSO	ELPSO
*f*_1_	6.72*E* + 03	1.23*E* + 04	9.99*E* + 03	1.43*E* + 03	8.82*E* + 03	9.19*E* + 03	8.97*E* + 03	1.69*E* + 01
*f*_2_	8.44*E* + 07	2.60*E* + 08	1.52*E* + 08	4.01*E* + 07	5.20*E* + 07	6.52*E* + 07	5.82*E* + 07	3.69*E* + 06
*f*_3_	6.54*E* + 03	8.45*E* + 03	7.87*E* + 03	4.01*E* + 02	5.13*E* + 03	6.46*E* + 03	5.86*E* + 03	3.74*E* + 02
*f*_4_	2.41*E* + 00	3.85*E* + 00	3.05*E* + 00	4.05*E* − 01	1.50*E* + 00	3.34*E* + 00	2.49*E* + 00	3.36*E* − 01
*f*_5_	6.29*E* + 02	8.32*E* + 02	7.06*E* + 02	7.66*E* + 01	2.33*E* + 02	2.78*E* + 02	2.54*E* + 02	4.92*E* + 01
*f*_6_	6.14*E* + 02	7.95*E* + 02	6.99*E* + 02	5.48*E* + 01	2.60*E* + 02	3.76*E* + 02	3.26*E* + 02	2.49*E* + 01
*f*_7_	3.88*E* + 02	2.38*E* + 03	1.45*E* + 03	5.36*E* + 02	1.56*E* + 04	1.64*E* + 04	1.58*E* + 04	1.53*E* + 02
*f*_8_	6.80*E* + 03	8.52*E* + 03	7.92*E* + 03	3.24*E* + 02	6.36*E* + 03	8.21*E* + 03	7.47*E* + 03	4.16*E* + 02
*f*_9_	2.50*E* + 01	3.71*E* + 05	1.81*E* + 04	6.70*E* + 03	1.61*E* + 03	8.03*E* + 03	5.37*E* + 02	2.24*E* + 01
*f*_10_	2.49*E* + 02	2.85*E* + 03	8.19*E* + 02	5.41*E* + 02	2.74*E* + 02	1.65*E* + 03	5.16*E* + 02	2.15*E* + 02
*f*_11_	1.89*E* + 03	6.01*E* + 03	3.83*E* + 03	1.05*E* + 03	3.76*E* + 03	7.64*E* + 03	5.66*E* + 03	8.50*E* + 02
*f*_12_	4.02*E* + 03	7.44*E* + 03	5.45*E* + 03	8.73*E* + 02	3.93*E* + 03	7.58*E* + 03	5.71*E* + 03	8.71*E* + 02

**Table 3:** Test results of 8 algorithms (*D* = 50).

B*e*nchmark function	PSO	MPSO	QPSO	IAP-PSO	*E*IW-PSO	CLPSO	SRPSO	*E*LPSO
*f*_1_	2.84*E* + 04	3.84*E* + 04	3.32*E* + 04	2.54*E* + 03	1.29*E* + 04	1.63*E* + 04	1.40*E* + 04	1.88*E* + 01
*f*_2_	3.44*E* + 08	6.21*E* + 08	4.82*E* + 08	7.03*E* + 07	1.58*E* + 08	2.93*E* + 08	2.15*E* + 08	1.00*E* + 07
*f*_3_	1.38*E* + 04	1.58*E* + 04	1.49*E* + 04	4.96*E* + 02	1.16*E* + 04	1.32*E* + 04	1.24*E* + 04	4.20*E* + 02
*f*_4_	3.49*E* + 00	4.58*E* + 00	4.03*E* + 00	2.54*E* + 00	4.34*E* + 00	3.62*E* + 00	4.83*E* − 01	2.80*E* − 01
*f*_5_	1.53*E* + 03	2.58*E* + 03	2.15*E* + 03	2.81*E* + 02	2.88*E* + 02	3.93*E* + 02	3.48*E* + 02	2.46*E* + 01
*f*_6_	1.61*E* + 03	2.37*E* + 03	1.98*E* + 03	2.32*E* + 02	6.17*E* + 02	7.64*E* + 02	7.01*E* + 02	3.93*E* + 01
*f*_7_	1.30*E* + 04	5.29*E* + 04	3.17*E* + 04	1.06*E* + 04	2.24*E* + 04	3.81*E* + 04	2.79*E* + 04	1.96*E* + 01
*f*_8_	2.90*E* + 03	5.19*E* + 03	3.96*E* + 03	8.14*E* + 02	3.08*E* + 03	3.32*E* + 03	3.21*E* + 03	2.97*E* + 02
*f*_9_	2.43*E* + 04	9.88*E* + 05	1.08*E* + 05	2.47*E* + 05	1.30*E* + 02	1.32*E* + 05	7.84*E* + 03	5.69*E* + 01
*f*_10_	8.21*E* + 02	3.79*E* + 03	2.05*E* + 03	7.09*E* + 02	4.81*E* + 03	2.04*E* + 03	9.86*E* + 02	6.31*E* + 02
*f*_11_	6.56*E* + 03	1.16*E* + 04	8.74*E* + 03	8.49*E* + 03	1.37*E* + 04	1.06*E* + 04	1.17*E* + 03	1.42*E* + 03
*f*_12_	7.24*E* + 03	1.41*E* + 04	1.07*E* + 04	1.79*E* + 03	9.00*E* + 03	1.68*E* + 04	1.18*E* + 04	1.71*E* + 03

**Table 4:** *T* test and Friedman test results of 8 algorithms.

	*f*_1_	*f*_2_	*f*_3_	*f*_4_	*f*_5_	*f*_6_	*f*_7_	*f*_8_	*f*_9_	*f*_10_	*f*_11_	*f*_12_	*w*/*t*/*l*	Rank mean
PSO	+	+	+	+	+	+	+	+	=	=	=	+	9/3/0	3.17
MPSO	+	+	+	=	+	=	+	=	+	=	−	+	7/4/1	3.08
QPSO	+	+	+	=	+	=	=	=	=	−	−	=	4/6/2	1.96
IAP-PSO	=	+	+	+	+	+	+	+	=	=	=	+	5/4/1	2.51
EIW-PSO	+	=	+	+	+	=	+	=	+	=	−	+	8/3/2	2.87
CLPSO	+	+	=	=	+	=	=	=	=	−	−	=	6/4/3	2.69
SRPSO	+	+	+	+	=	+	=	+	=	=	=	+	7/2/9	1.87
ELPSO														1.79

**Table 5:** Wilcoxon rank test results of 8 algorithms.

Dimension	ELPSO VS	*p*-value	+	≈	−	*α*=0.05
10	PSO	0.005173	10	0	0	Yes
	MPSO	0.007182	10	0	0	Yes
	QPSO	0.006253	9	1	0	Yes
	IAP-PSO	0.004179	10	0	0	Yes
	EIW-PSO	0.005187	10	1	0	Yes
	CLPSO	0.005462	10	0	0	Yes
	SRPSO	0.005383	10	0	0	Yes

30	PSO	0.005875	10	0	0	Yes
	MPSO	0.006217	10	1	0	Yes
	QPSO	0.006348	10	0	0	Yes
	IAP-PSO	0.005981	10	0	0	Yes
	EIW-PSO	0.006312	10	0	0	Yes
	CLPSO	0.005819	10	1	0	Yes
	SRPSO	0.006416	10	0	0	Yes

50	PSO	0.005349	10	0	0	Yes
	MPSO	0.007381	10	0	0	Yes
	QPSO	0.006945	10	0	0	Yes
	IAP-PSO	0.005946	10	0	0	Yes
	EIW-PSO	0.006218	10	1	0	Yes
	CLPSO	0.006325	10	0	0	Yes
	SRPSO	0.005419	10	0	0	Yes

**Table 6:** Average number of iterations under the specified accuracy.

	*f*_1_	*f*_2_	*f*_3_	*f*_4_	*f*_5_	*f*_6_	*f*_7_	*f*_8_	*f*_9_	*f*_10_	*f*_11_	*f*_12_
PSO	—	243	—	—	—	—	—	—	227	—	204	—
MPSO	164	150	213	94	—	113	—	208	186	94	125	194
QPSO	62	18	102	22	179	61	62	98	151	42	149	83
IAP-PSO	87	54	115	96	49	117	103	115	147	98	99	96
EIW-PSO	114	71	79	83	65	49	58	92	83	30	74	60
CLPSO	79	85	105	92	71	72	64	84	101	53	59	86
SRPSO	34	64	86	78	48	63	55	71	45	44	39	67
ELPSO	11	12	16	14	34	9	10	16	21	19	24	13

**Table 7:** Comparison of prediction error indexes of model prediction error index comparison.

Prediction model	MAE	RMSE
BP	0.0745	0.0895
LSTM	0.0174	0.0186
BiGRU	0.0148	0.0156
ELPSO-BiGRU	0.0082	0.0108
EMD-ELPSO-BiGRU	0.0032	0.0054

**Table 8:** Comparison of prediction absolute of different prediction models at each time point.

Serial number	BP	LSTM	BiGRU	ELPSO-BiGRU	EMD-ELPSO-BiGRU
1	0.01014	0.09067	0.0056	0.00266	0.00069
2	0.00791	0.06355	0.00547	0.00211	0.00254
3	0.00803	0.08235	0.00707	0.00213	0.00290
4	0.01153	0.08235	0.00707	0.00213	0.00116
5	0.01041	0.10887	0.00496	0.00377	0.00037
6	0.01006	0.10584	0.0058	0.00443	0.00017
7	0.01043	0.02663	0.00674	0.00209	0.00051
8	0.00904	0.03808	0.00634	0.00223	0.00071
9	0.00026	0.00825	0.01207	0.00047	0.00205
10	0.00849	0.05474	0.00904	0.00075	0.00014
11	0.00268	0.01946	0.00955	0.00883	0.00331
12	0.00733	0.0477	0.01169	0.02419	0.00343
13	0.00483	0.09163	0.00756	0.00587	0.00276
14	0.00918	0.15478	0.0063	0.00414	0.00157
15	0.00989	0.12663	0.00424	0.00485	0.00211
16	0.00909	0.03466	0.00768	0.00258	0.00045
17	0.00875	0.0201	0.00778	0.00035	0.00107
18	0.00116	0.07084	0.01005	0.01816	0.03832

## Data Availability

The datasets generated during and/or analyzed during the current study are available from the corresponding author on reasonable request.
